# Targeting Beta-Amyloid at the CSF: A New Therapeutic Strategy in Alzheimer’s Disease

**DOI:** 10.3389/fnagi.2018.00100

**Published:** 2018-04-16

**Authors:** Manuel Menendez-Gonzalez, Huber S. Padilla-Zambrano, Gabriel Alvarez, Estibaliz Capetillo-Zarate, Cristina Tomas-Zapico, Agustin Costa

**Affiliations:** ^1^Servicio de Neurologia, Hospital Universitario Central de Asturias, Oviedo, Spain; ^2^Department of Cellular Morphology and Biology, University of Oviedo, Oviedo, Spain; ^3^Instituto de Investigacion Sanitaria del Principado de Asturias, Oviedo, Spain; ^4^Centro de Investigaciones Biomedicas (CIB), University of Cartagena, Cartagena, Colombia; ^5^HealthSens, S.L., Oviedo, Spain; ^6^Departamento de Neurociencias, Universidad del Pais Vasco (UPV/EHU), Leioa, Spain; ^7^El Centro de Investigación Biomédica en Red sobre Enfermedades Neurodegenerativas, Madrid, Spain; ^8^Achucarro Basque Center for Neuroscience, Leioa, Spain; ^9^Ikerbasque, Basque Foundation for Science, Bilbao, Spain; ^10^Department of Functional Biology, University of Oviedo, Oviedo, Spain; ^11^Department of Physical and Analytical Chemistry, University of Oviedo, Oviedo, Spain

**Keywords:** Alzheimer disease, amyloid beta-peptides, cerebrospinal fluid, immunotherapy, “CSF sink hypothesis”

## Abstract

Although immunotherapies against the amyloid-β (Aβ) peptide tried so date failed to prove sufficient clinical benefit, Aβ still remains the main target in Alzheimer’s disease (AD). This article aims to show the rationale of a new therapeutic strategy: clearing Aβ from the CSF continuously (the “CSF-sink” therapeutic strategy). First, we describe the physiologic mechanisms of Aβ clearance and the resulting AD pathology when these mechanisms are altered. Then, we review the experiences with peripheral Aβ-immunotherapy and discuss the related hypothesis of the mechanism of action of “peripheral sink.” We also present Aβ-immunotherapies acting on the CNS directly. Finally, we introduce alternative methods of removing Aβ including the “CSF-sink” therapeutic strategy. As soluble peptides are in constant equilibrium between the ISF and the CSF, altering the levels of Aβ oligomers in the CSF would also alter the levels of such proteins in the brain parenchyma. We conclude that interventions based in a “CSF-sink” of Aβ will probably produce a steady clearance of Aβ in the ISF and therefore it may represent a new therapeutic strategy in AD.

## Physiological Clearance of Aβ

Amyloid beta (Aβ) denotes peptides of 36–43 amino acids that are intrinsically unstructured, meaning that in solution it does not acquire a unique tertiary fold but rather populates a set of structures. These peptides derive from the amyloid precursor protein (APP), which is cleaved by beta- (BACE) and gamma-secretases to yield Aβ (Menendez-Gonzalez et al., 2005; O’Nuallain et al., 2010).

Amyloid beta is cleared from the brain by several independent mechanisms (Malm et al., 2010; Diem et al., 2017; Zuroff et al., 2017), including drainage to the vascular and glymphatic systems (DeMattos et al., 2001; Iliff et al., 2012, 2013; Tarasoff-Conway et al., 2015; Bakker et al., 2016; Zuroff et al., 2017), and *in situ* degradation by glial cells (Ries and Sastre, 2016; Zuroff et al., 2017). Astrocytes and microglia can produce Aβ degrading proteases like neprilysin, as well as chaperones involved in the clearance of Aβ. There is also a receptor mediated endocytosis, where receptors located in the surface of glial cells are involved in the uptake and clearance of Aβ, like lipoprotein receptor-related protein 1 (LRP), receptor for advanced glycation end products (RAGE) and others (Ries and Sastre, 2016). In transcytosis, Aβ is removed from ISF across the blood brain barrier (BBB) by LRP (Yamada et al., 2009). LRP binds Aβ in the brain and then transports it across the BBB into the systemic blood. The LRP extracellular domain is cleaved allowing the LRP bound to Aβ. RAGE protein brings unbound Aβ back into the CNS. The whole process is regulated by PICALM (Zhao et al., 2015). A perivascular pathway facilitates CSF flow through the brain parenchyma and the clearance of interstitial solutes, including Aβ (Iliff et al., 2012, 2013). It was thought that changes in arterial pulsatility may contribute to accumulation and deposition of toxic solutes, including Aβ, in the aging brain (Iliff et al., 2012, 2013). However, mathematical simulation showed that arterial pulsations are not strong enough to produce drainage velocities comparable to experimental observations and that a valve mechanism such as directional permeability of the intramural periarterial drainage pathway is necessary to achieve a net reverse flow (Diem et al., 2017).

## Altered Clearance of Aβ in Alzheimer’s Disease

The pathophysiology of Alzheimer’s disease (AD) is characterized by the accumulation of Aβ and phospho-tau protein in the form of neuritic plaques and neurofibrillary tangles, respectively (Braak and Braak, 1991; Atwood et al., 2002). Aβ molecules can aggregate to form flexible soluble oligomers, which exist in several forms and are toxic to neurons (Haass and Selkoe, 2007), and finally into diffuse and dense plaques. Moreover, variable amounts of misfolded oligomers (known as “seeds”) are taken up by neurons then transmitted from neuron to neuron via the extracellular milieu and can propagate aggregates by a ‘seeding’ or “prion like” mechanism (Walker et al., 2016; Lei et al., 2017). Tau also forms such prion-like misfolded oligomers, and there is some evidence that misfolded Aβ can induce tau misfolding (Pulawski et al., 2012; Nussbaum et al., 2013).

Amyloid-β accumulation has been hypothesized to result from an imbalance between Aβ production and clearance. An overproduction is probably the main cause of the disease in the familial AD where a mutation in the *APP, PSEN1*, or *PSEN2* genes is present (presenilins are postulated to regulate APP processing through their effects on gamma-secretase) while altered clearance is probably the main cause of the disease in sporadic AD. A good amount of studies reporting altered clearance of Aβ in AD have been published in recent years (Atwood et al., 2002; Mawuenyega et al., 2010; Tarasoff-Conway et al., 2015; Ries and Sastre, 2016; de Leon et al., 2017; Zuroff et al., 2017), becoming one of the “hot-topics” in AD research today.

The different clearance systems probably contribute to varying extents on Aβ homeostasis. Any alteration to their function may trigger the progressive accumulation of Aβ (Morrone et al., 2015; Tarasoff-Conway et al., 2015; de Leon et al., 2017), which is the fundamental step in the hypothesis of the amyloid cascade (Lambert et al., 1998; Quan and Banks, 2007; Mawuenyega et al., 2010; Bateman et al., 2012; Fagan et al., 2014; Fleisher et al., 2015). There is a relationship between the decrease in the rate of turnover of amyloid peptides and the probability of aggregation due to incorrect protein misfolding (Patterson et al., 2015) resulting in its accumulation. As soluble molecules can move in constant equilibrium between the ISF and the CSF, Aβ monomers and oligomers can be detected in the CSF. The AP42, and Aβ oligomer/protofibril levels in cortical biopsy samples are higher in subjects with insoluble cortical Aβ aggregates than in subjects without aggregates, and brain tissue levels of AP42 are negatively correlated with CSF AP42 (Patel et al., 2012; Cesarini and Marklund, 2018). Indeed, measuring the levels of Aβ in the CSF is one of the main proposed biomarkers already accepted in the diagnostic criteria of AD (McKhann et al., 2011). It has been reported that levels of Aβ in the CSF vary with time. Results from cross-sectional analysis in familial AD demonstrate higher levels of Aβ in the CSF from mutation carriers compared to controls very early in the disease process (∼20–30 years prior to estimated symptom onset), which then drop with disease progression, becoming significantly lower than controls ∼10–20 years prior to symptom onset (Morrone et al., 2015; Tarasoff-Conway et al., 2015). These low levels then begin to plateau with the development of cognitive symptoms (Iliff et al., 2013). In sporadic AD at very early preclinical stage (transitional stage) there might be either elevations or reductions in CSF AP42 (Clark et al., 2018; de Leon et al., 2018).

## Therapeutic Clearance of Aβ

Different approaches have been investigated with the aim of removing brain Aβ. Decreasing Aβ production might be the first approach that one can think of to reduce ISF Aβ. For instance, the inhibition BACE is one of the first therapeutic strategies formulated after the amyloid cascade hypothesis, and it is still being explored today. Alternatively, increasing the elimination of Aβ by enzymatic degradation or by clearance enhancement may be able to slow down both the aggregation and the spread processes of the disease given the relevance of Aβ as a substrate in AD (Ryan et al., 2010). Among all strategies to enhance the clearance of Aβ, immunotherapy is the most explored approach so far.

### Aβ Immunotherapy

#### Peripheral Aβ Immunotherapy and the Mechanism of Action of “Peripheral-Sink”

The Aβ immunotherapy consist on activating the immune system against Aβ through the induction (active immunotherapy) or administration (passive immunotherapy) of Aβ-antibodies (Menendez-Gonzalez et al., 2011). Passive immunotherapy can be either monoclonal (mAbs) or polyclonal (immunoglobulins). Active immunization activates the immune system to produce specific antigen antibodies. In AD, Aβ or fragments of Aβ can be used as an antigen, conjugated to a T-cell epitope-bearing protein, together with a booster of the immune system (adjuvant). Passive immunization avoids the need to activate and initiate an immune response to produce antigen-specific antibodies. In both active and passive immunization, Aβ-antibodies bind to Aβ, potentially promoting the clearance of the peptide (Georgievska et al., 2015).

Some interventions have been shown to produce some positive changes on brain Aβ, both in animal models and in human subjects. Unfortunately, these neuropathological benefits were not accompanied by sufficient clinical benefit; therefore, none of these therapies have been transferred to the clinic. One of the reasons may be that effective development of AD therapeutic strategies targeting Aβ require very early administration (before amyloid-plaques are in place) and consideration of the age- and ApoE-specific changes to endogenous Aβ clearance mechanisms in order to optimize efficacy (Morrone et al., 2015).

Understanding how Aβ-antibodies remove Aβ from the brain is a key in the design of Aβ immunotherapies for AD. Two distinct but not mutually exclusive mechanisms have been proposed: The “microglial phagocytosis” would require the antibodies to enter the brain, where they mediate the uptake of Aβ into local microglia. The “peripheral sink” mechanism of action relies only on peripheral antibodies to sequester Aβ in the systemic blood, lowering the level of free Aβ and inducing the brain to release its store of the peptide. This sequestration of circulating Aβ produces a shift in the concentration gradient of Aβ between the brain and the blood causing an efflux of Aβ out of the brain. Thus, it has been hypothesized that reducing Aβ peptides in the periphery would be a way to diminish Aβ levels and plaque load in the brain (Xiang et al., 2015). However, controversy still remains, with evidence both in favor and against the peripheral sink mechanism (Deane et al., 2009; Yamada et al., 2009). Studies with transgenic AD mice seem to validate the hypothesis of the peripheral sink as the main mechanism of Aβ removal after immunization. Some others showed that little or no antibody enters the brain (Vasilevko et al., 2007) and that peripheral anti-Aβ antibody alters CNS and plasma Aβ clearance decreasing brain Aβ burden (DeMattos et al., 2001). Additionally, mice with the Dutch and Iowa mutations have an Aβ peptide that is a poor substrate for the efflux transporter LRP, and so accumulates to high levels in the brain. Indeed, these mice have no peripheral sink effect, and despite a massive buildup of vascular amyloid and parenchymal plaque in brain, Aβ remains undetectable in their blood (Deane et al., 2004; Davis et al., 2006). Direct measurements of brain extracts revealed that little or no antibody was able to enter the brain from the periphery (Ryan et al., 2010). Sagare et al. (2007) showed that infusing in the blood a recombinant version of LRP (sLRP) binding Aβ lowers plaque burden in these mice, producing the peripheral sink effect. Authors also proved that Aβ shifted out of the CNS into the blood (Sagare et al., 2007).

On the other hand, sustained peripheral depletion of Aβ with a new form of neprilysin, which fuses with albumin to prolong plasma half-life, is designed to confer increased Aβ degradation activity and does not affect central Aβ levels in transgenic mice, rats and monkeys (Henderson et al., 2014). In other report (Deane et al., 2009), authors tested the peripheral sink hypothesis by investigating how selective inhibition of Aβ production in the periphery, using a BACE inhibitor or reducing BACE gene dosage, affects Aβ load in the brain. Selective inhibition of peripheral BACE activity in wildtype or transgenic mice reduced Aβ levels in the periphery but not in the brain, even after chronic treatment over several months. In contrast, a BACE inhibitor with improved brain disposition reduced Aβ levels in both brain and periphery already after acute dosing. BACE heterozygous mice displayed an important reduction in plasma Aβ, whereas Aβ reduction in the brain was much lower. These data suggest that reduction of Aβ in the periphery is not sufficient to reduce brain Aβ levels and that BACE is not the rate-limiting enzyme for Aβ processing in the brain (Georgievska et al., 2015). Recent research suggests that CSF naturally occurring antibodies against Aβ seem to have a protective effect for AD, while serum naturally occurring antibodies against Aβ do not seem to have any effect (Kimura et al., 2017; Menendez Gonzalez, 2017a). In line with this, Piazza et al. (2013) reported the first evidence about the participation of natural anti-Aβ autoantibodies in cerebral amyloid-related angiopathy (CAA) and the possible elimination mechanism of soluble Aβ in the CSF by antibodies. Today, CSF anti-Aβ autoantibodies are known to play a key role in the development of amyloid-related imaging abnormalities (ARIA) (DiFrancesco et al., 2015; Chen et al., 2016; Piazza and Winblad, 2016), which are MRI signal changes representing vasogenic edema (VE) and microhemorrhages (mH). VE and mH share some common underlying pathophysiological mechanisms, both in the natural history of AD and in immunotherapies (Sperling et al., 2011). Furthermore, this ARIA has been associated with a massive release of soluble Aβ, plaques and vascular deposits during the acute inflammatory phase (DiFrancesco et al., 2015; Chen et al., 2016; Piazza and Winblad, 2016).

Administered monoclonal antibodies also showed molecular effect, but clinical benefit in humans was not significant. For instance, Solanezumab increases the elimination of soluble Aβ and decreases the deposition of cerebral amyloid plaque in AD mice. In clinical trials, the administration of Solanezumab in patients with mild to moderate AD generated an increase of unbound Aβ in CSF, suggesting that the antibody has a direct peripheral effect with central indirect effect. However, clinical trials showed not improvement of the cognitive and functional capacities of patients (Doody et al., 2014; Chen et al., 2016; Siemers et al., 2016). Similarly, Bapineuzumab modifies Aβ accumulation and CSF biomarkers, but none of the trials showed a significant clinical benefit (Salloway et al., 2014).

#### Aβ-Immunotherapy Into the CNS

Many investigators have indicated that peripheral clearance through the BBB is not recommended in elderly people, in whom the normal transport of Aβ may present alterations. In addition, the risk of antibody-mediated hemorrhage in sites of cerebral amyloid angiopathy decreases the authors’ interest in peripheral passive as well as in active reduction mediated by CNS Aβ antibodies. Due to this, it has been considered that the direct administration of immunotherapy to the CNS is more efficient than the peripheral one, but the intrinsic characteristics of the BBB make the pharmacological approach difficult. This has led to the search for strategies to overcome the BBB. These approaches were divided into two categories: the first comprises techniques that facilitate the passage of drugs through the BBB (for example, molecular “Trojan horses,” oligopeptides transporters coupled to protons, exosomes, liposomes, nanoparticles, chimeric peptides, prodrugs); and the second consists on techniques that avoid BBB through direct delivery to the SNC. In this last category, the techniques have been investigated include the interruption of BBB (for example, with ultrasound and microbubbles) and intrathecal, intracerebroventricular and intranasal administration (Wilcock et al., 2003; Carty et al., 2006). Although much less explored, passive Aβ-immunotherapy into the CNS has been tested on animal models. Several groups have reported to have achieved clearance of brain Aβ after intracerebral or intraventricular injection of either Aβ antibodies (Wilcock et al., 2003, 2004; Oddo et al., 2004; Carty et al., 2006; Levites et al., 2006), antibodies to oligomeric assemblies of Aβ (Chauhan, 2007) or promoting cellular expression of Aβ-specific antibodies, delivered using viral vectors (Ryan et al., 2010). In most cases, the clearance was rapid (within a few days), but the benefits of the injections were transient because the decrease in amyloid plaques approached reversion at 30 days (Sevigny et al., 2016). Authors also observed a decrease in tau hyperphosphorylation, an increase in the number of microglia counts and an improved learning behavior (Doody et al., 2014). In different reports, the level of clearance achieved by this method varies significantly and ranges from what appears to be elimination throughout the CNS (Sakai et al., 2016) to the limited elimination of diffuse amyloid around the site of antibody injection (Ostrowitzki et al., 2012).

Some human monoclonal antibodies have been shown to enter the brain even when administered peripherally. In a transgenic mouse model of AD, Aducanumab is shown to enter the brain, bind parenchymal Aβ, and reduce soluble and insoluble Aβ in a dose-dependent manner. In patients with prodromal or mild AD, 1 year of monthly intravenous infusions of Aducanumab reduces brain Aβ in a dose- and time-dependent manner. This is accompanied by a slowing of clinical decline. The main safety issues are amyloid-related imaging abnormalities (Sevigny et al., 2016). Phase 3 clinical trials are ongoing. Gantenerumab preferentially interacts with aggregated brain Aβ, both parenchymal and vascular. This antibody acts centrally to disassemble and degrade amyloid plaques by recruiting microglia and activating phagocytosis (Ostrowitzki et al., 2012) but it does not alter plasma Aβ (Bohrmann et al., 2012). As with Adenacumab, trials showed positive trends in clinical scales, main safety worries are amyloid-related imaging abnormalities and clinical trials in different phases are ongoing.

In conclusion, no Aβ immunotherapy has demonstrated significant efficacy in humans to date. A meta-analysis of immunotherapies (Penninkilampi et al., 2017) found no significant treatment differences for typical primary outcome measures. Clinical benefits of peripheral immunotherapy in humans are limited, while the benefits of central immunotherapy in animal models are transient.

### Alternative Therapeutic Strategies

Despite Aβ immunotherapy showed not conclusive results to date, Aβ remains the main target in AD. A study using an image biomarker determined that a 15% decrease in Aβ is related to a cognitive improvement of 15–20% (Liu et al., 2015). For all that, there is an urgent need to find alternative methods to achieve a depletion of Aβ in the brain.

**FIGURE 1:**
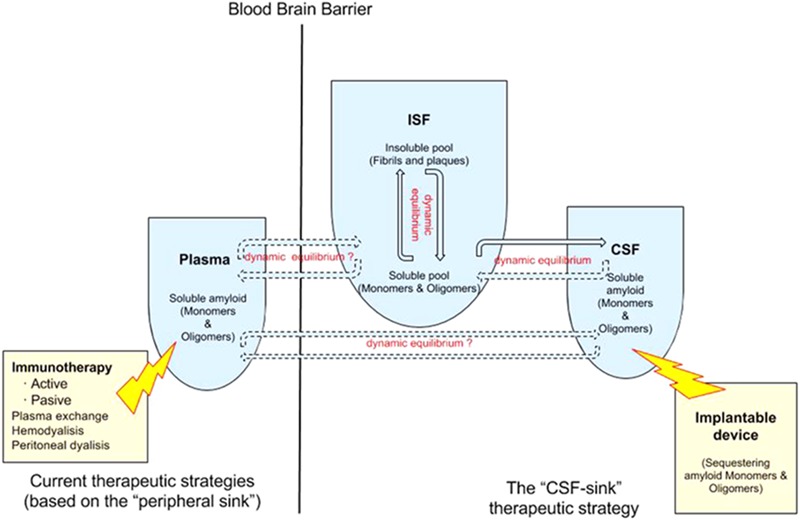
Double dynamic equilibrium of Aβ: there is a bidirectional equilibrium between insoluble and soluble pools of Aβ in the ISF and there is a second equilibrium, also probably bidirectional, of soluble Aβ between the ISF and the CSF. The “CSF-sink therapeutic strategy” consists on sequestering target proteins from the CSF with implantable devices, thus inducing changes in the levels of these proteins in the ISF. Current therapeutic strategies rely on the “peripheral sink” hypothesis mostly. There is some controversy about the existence of a equilibrium of Aβ between plasma and the ISF/CSF.

A number of studies showed that blood dialysis and plasmapheresis reduces Aβ levels in plasma and CSF in humans and attenuates AD symptoms and pathology in AD mouse models (58,6165), suggesting that removing Aβ from the plasma seems to be an effective -albeit indirect- way of removing Aβ. Different methodologies, from peritoneal dialysis (Jin et al., 2017) to hemodialysis (Kitaguchi et al., 2015; Sakai et al., 2016; Tholen et al., 2016) and plasma exchange (Boada et al., 2009), reported some extent of success removing Aβ from the plasma, which in turn reduces Aβ in the CSF and in the ISF -this last compartment has been confirmed in animals only-. Again, the “peripheral-sink hypothesis” adds new sources of support from these alternative strategies (**Figure 1**).

However, there might be a much more direct way of removing Aβ from the ISF than clearing it from the plasma: clearing it from the CSF. A starting rationale is that there is an equilibrium of Aβ levels between the ISF and plasma in AD transgenic mice before developing Aβ deposits (DeMattos et al., 2002; Cirrito et al., 2003; Hong et al., 2011; Nag et al., 2011). However, this equilibrium is lost once Aβ deposits are in place while the equilibrium of Aβ between the ISF and the CSF still persists (DeMattos et al., 2002). Some studies also found a relationship between the load of cortical deposits and levels in the CSF in humans who underwent neurosurgery (ventriculo-peritoneal shunt) (Seppala et al., 2012; Pyykko et al., 2014; Herukka et al., 2015; Abu Hamdeh et al., 2018). At equilibrium, Aβ remains predominantly monomeric up to 3 pM, above which forms large aggregates (Nag et al., 2011). Once aggregated are in place, amyloid deposits can rapidly sequester soluble A from the ISF (Cirrito et al., 2003; Hong et al., 2011). Aβ in the ISF in plaque-rich mice is thought to be derived not from new A biosynthesis but rather from the large reservoir of less soluble Aβ in brain parenchyma (Cirrito et al., 2003). Moreover, a portion of the insoluble Amyloid pool is in dynamic equilibrium with ISF Amyloid. *In vitro* studies have shown that A aggregates contain a readily dissociable pool of Aβ, or “docked Aβ” as well as a long-lasting or stable “locked” pool of Aβ (Maggio et al., 1992; Esler et al., 2000). *In vitro*, as the concentration of Aβ in solution decreases, this docked pool can quickly dissociate from fibrils. *In vivo*, when Aβ production is inhibited and ISF Aβ levels begin to decrease, it is likely that this associated docked pool can return to solution over a finite period of time, as occurs *in vitro*, causing this pool of Aβ to dissociate from fibrils and become soluble. This results in a prolonged apparent half-life of ISF Aβ in animals with Aβ deposition (Cirrito et al., 2003).

**FIGURE 2:**
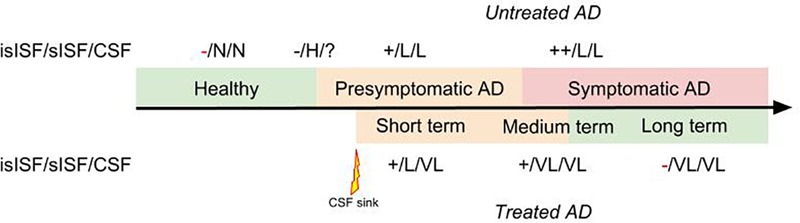
Diagram representing the therapeutic effect of a “CSF-sink” intervention on the predicted levels of Aβ in the insoluble ISF (isISF), soluble ISF (sISF) and CSF pools in a patient with AD treated at presymptomatic stage. Legend: +, positive deposits; -, negative deposits; N, normal; H, high; VH, very high; L, low; VL, very low.

We previously posed the hypothesis that soluble proteins can be cleared from the brain with interventions where soluble proteins are continuously removed from the CSF (Liu et al., 2015; Menendez Gonzalez, 2017c). This is since soluble proteins are in constant equilibrium between the ISF and the CSF. Therefore, clearing Aβ from the CSF continuously will probably promote the efflux of Aβ from the ISF to the CSF (**Figure 2**).

The “CSF-sink” therapeutic strategy consists on sequestering Aβ from the CSF (**Figure 2**). Today, we can conceive several ways of accessing the CSF with implantable devices (Menendez Gonzalez, 2017b). These devices can be endowed with different technologies able to capture target molecules, such as Aβ, from the CSF. Thus, these interventions would work as a central sink of Aβ, reducing the levels of CSF Aβ, and by means of the CSF-ISF equilibrium would promote the efflux of Aβ from the ISF to the CSF (**Figure 2**).

**FIGURE 3:**
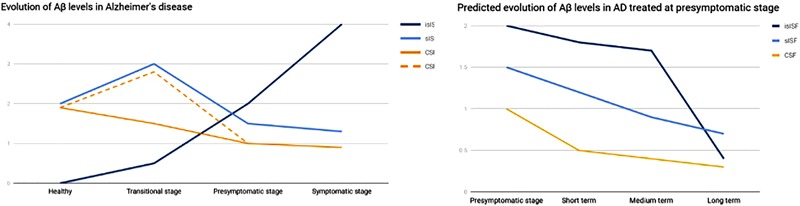
Graph representing the evolution of Aβ in the different pools. On the left, evolution of Aβ levels across the different stages of a AD. On the right, predicted evolution of Aβ levels in a case of AD treated at presymptomatic stage. islSF, insoluble pool in the ISF; slSF, soluble pool in the ISF.

A study on the Aβ clearance kinetics suggests that the speed and efficiency of Aβ clearance pathways may influence the effect on Aβ deposits (Yuede et al., 2016). A therapeutic strategy aimed at rapid clearance at only high concentrations may be different from a strategy that is designed for a sustained, possibly larger, suppression of Aβ. The “CSF-sink” therapeutic strategy is expected to provide an intense and sustained depletion of Aβ in the CSF and, in turn, a steady decrease Aβ in the ISF, preventing the formation of new aggregates and deposits in the short term and potentially reversing the already existing deposits in the medium and long terms (**Figure 3**).

Albeit AD is a complex disease, and targeting one single molecule might not be enough to hinder the whole neurodegenerative process, we consider this strategy is worth trying, since it is feasible and potentially efficient.

Finally, we would like to mention this strategy might also be valid for other neurodegenerative and neuroimmune diseases where target molecules are well identified and present in the CSF in equilibrium with the ISF. A series of studies in cellular and animal models are needed to prove this hypothesis.

## Conclusion

We introduce the rationale basis for the “CSF-sink” hypothesis and conclude that continuous depletion of Aβ in the CSF will probably produce a steady clearance of Aβ in the ISF. Implantable devices aimed at sequestering Aβ from the CSF may represent a new therapeutic strategy in AD.

## Author Contributions

MM-G is the author of the hypothesis and wrote the whole manuscript. All the other authors revised the existing literature and critically reviewed the manuscript.

## Conflict of Interest Statement

GA is employed by HealthSens, S.L. The other authors declare that the research was conducted in the absence of any commercial or financial relationships that could be construed as a potential conflict of interest.
